# Conferencia sobre Cambio Climático COP27: se necesita una acción urgente para África y el mundo^*^

**DOI:** 10.26633/RPSP.2022.215

**Published:** 2022-11-15

**Authors:** Lukoye Atwoli, Gregory E. Erhabor, Aiah A. Gbakima, Abraham Haileamlak, Jean-Marie Kayembe Ntumba, James Kigera, Laurie Laybourn-Langton, Bob Mash, Joy Muhia, Fhumulani Mavis Mulaudzi, David Ofori-Adjei, Friday Okonofua, Arash Rashidian, Maha El-Adawy, Siaka Sidibé, Abdelmadjid Snouber, James Tumwine, Mohammad Sahar Yassien, Paul Yonga, Lilia Zakhama, Chris Zielinski

**Affiliations:** 1 Editor jefe, East African Medical Journal Editor jefe, East African Medical Journal; 2 Editor jefe, West African Journal of Medicine Editor jefe, West African Journal of Medicine; 3 Editor jefe, Sierra Leone Journal of Biomedical Research Editor jefe, Sierra Leone Journal of Biomedical Research; 4 Editor jefe, Ethiopian Journal of Health Sciences Editor jefe, Ethiopian Journal of Health Sciences; 5 Editor jefe, Annales Africaines de Medecine Editor jefe, Annales Africaines de Medecine; 6 Editor jefe, Annals of African Surgery Editor jefe, Annals of African Surgery; 7 University of Exeter University of Exeter; 8 Editor jefe, African Journal of Primary Health Care & Family Medicine Editor jefe, African Journal of Primary Health Care & Family Medicine; 9 London School of Medicine and Tropical Hygiene London School of Medicine and Tropical Hygiene; 10 Editor jefe, Curationis Editor jefe, Curationis; 11 Editor jefe, Ghana Medical Journal Editor jefe, Ghana Medical Journal; 12 Editor jefe, African Journal of Reproductive Health Editor jefe, African Journal of Reproductive Health; 13 Editor jefe, Eastern Mediterranean Health Journal Editor jefe, Eastern Mediterranean Health Journal; 14 Director de promoción de la salud, Eastern Mediterranean Health Journal Director de promoción de la salud, Eastern Mediterranean Health Journal; 15 Director de publicación, Mali Médical Director de publicación, Mali Médical; 16 Editor gerente, Journal de la Faculté de Médecine d’Oran Editor gerente, Journal de la Faculté de Médecine d’Oran; 17 Editor jefe, African Health Sciences Editor jefe, African Health Sciences; 18 Editor jefe, Evidence-Based Nursing Research Editor jefe, Evidence-Based Nursing Research; 19 Editor gerente, East African Medical Journal Editor gerente, East African Medical Journal; 20 Editor jefe, La Tunisie Médicale Editor jefe, La Tunisie Médicale; 21 University of Winchester University of Winchester

## LAS NACIONES RICAS DEBEN AUMENTAR SU APOYO A ÁFRICA Y A LOS PAÍSES VULNERABLES PARA HACER FRENTE A LOS IMPACTOS PASADOS, PRESENTES Y FUTUROS DEL CAMBIO CLIMÁTICO

El informe de 2022 del Grupo Intergubernamental de Expertos sobre el Cambio Climático (IPCC) dibuja un oscuro panorama sobre el futuro de la vida en la Tierra, caracterizado por el colapso de los ecosistemas, la extinción de especies y los riesgos climáticos, como las olas de calor y las inundaciones (1). Todos ellos están relacionados con problemas de salud física y mental, con consecuencias directas e indirectas de aumento de la morbilidad y la mortalidad. Para evitar estos efectos catastróficos para la salud en todas las regiones del planeta, existe un amplio acuerdo –como sostuvieron 231 revistas de salud en 2021– en que el aumento de la temperatura global debe limitarse a menos de 1,5 °C en comparación con los niveles preindustriales.

Aunque el Acuerdo de París de 2015 esboza un marco de acción global que incorpora la provisión de financiación climática a los países en desarrollo, este apoyo aún no se ha materializado (2). La COP27 es la quinta Conferencia de las Partes que se organiza en África desde su creación en 1995. En vísperas de esta reunión, nosotros –como editores de revistas de salud de todo el continente– hacemos un llamado a la acción urgente para garantizar que sea la COP la que finalmente haga justicia climática a África y a los países vulnerables. Esto es esencial no sólo para la salud de esos países, sino para la salud de todo el mundo.

## ÁFRICA HA SUFRIDO DE FORMA DESPROPORCIONADA, AUNQUE HA HECHO POCO PARA PROVOCAR LA CRISIS

La crisis climática ha repercutido en los determinantes ambientales y sociales de la salud en toda África, provocando efectos sanitarios devastadores (3). Los impactos sobre la salud pueden ser consecuencia directa de las perturbaciones medioambientales e indirectamente a través de los efectos socialmente mediados (4). Los riesgos relacionados con el cambio climático en África son inundaciones, sequía, olas de calor, reducción de la producción de alimentos y disminución de la productividad laboral (5).

Las sequías en el África subsahariana se han triplicado entre 1970 y 1979 y entre 2010 y 2019 (6). En 2018, ciclones devastadores afectaron a 2,2 millones de personas en Malawi, Mozambique y Zimbabue (6). En África occidental y central, graves inundaciones provocaron mortalidad y migraciones forzadas por la pérdida de techo, tierras cultivadas y ganado (7). Los cambios en la ecología de los vectores provocados por las inundaciones y los daños en la higiene ambiental han provocado un aumento de las enfermedades en toda el África subsahariana, con incrementos en la malaria, el dengue, la fiebre de Lassa, la fiebre del Valle del Rift, la enfermedad de Lyme, el virus del Ébola, el virus del Nilo Occidental y otras infecciones (8, 9). El aumento del nivel del mar reduce la calidad del agua, lo que provoca enfermedades transmitidas por el agua, incluidas las enfermedades diarreicas, una de las principales causas de mortalidad en África (8). Las condiciones meteorológicas extremas dañan el agua y el suministro de alimentos, aumentando la inseguridad alimentaria y la malnutrición, causando 1,7 millones de muertes al año en África (10). Según la Organización de las Naciones Unidas para la Agricultura y la Alimentación, la malnutrición ha aumentado casi un 50% desde 2012, debido al papel central que desempeña la agricultura en las economías africanas (11). Las perturbaciones ambientales y sus efectos en cadena también causan graves daños a la salud mental (12). En total, se calcula que la crisis climática ha destruido una quinta parte del producto interior bruto de los países más vulnerables a las perturbaciones climáticas (13).

Los daños sufridos por África deberían preocupar sobremanera a todas las naciones. Esto se debe en parte a razones morales. Es muy injusto que las naciones más afectadas sean las que menos han contribuido a las emisiones globales acumuladas, que son las que impulsan la crisis climática y sus efectos cada vez más graves. América del Norte y Europa han aportado el 62% de las emisiones de dióxido de carbono desde la Revolución Industrial, mientras que África sólo ha contribuido con el 3% (14).

## LA LUCHA CONTRA LA CRISIS CLIMÁTICA NECESITA DE TODAS LAS MANOS

Sin embargo, no es sólo por razones morales por lo que todas las naciones deberían preocuparse por África. Los impactos agudos y crónicos de la crisis climática crean problemas como pobreza, enfermedades infecciosas, migraciones forzadas y conflictos que se propagan a través de los sistemas globalizados (6, 15). Estas repercusiones afectan a todas las naciones. La COVID-19 sirvió de llamada de atención sobre estas dinámicas globales, y no es casualidad que los profesionales de la salud hayan participado activamente en la identificación y respuesta a las consecuencias de los crecientes riesgos sistémicos para la salud. Pero las lecciones de la pandemia de COVID-19 no deben limitarse al riesgo pandémico (16, 17). Por el contrario, es imperativo que el sufrimiento de las naciones de primera línea, incluidas las de África, sea la consideración principal en la COP27: en un mundo interconectado, dejar a los países a merced de las perturbaciones medioambientales crea una inestabilidad que tiene graves consecuencias para todas las naciones.

El objetivo principal de las cumbres sobre el clima sigue siendo reducir rápidamente las emisiones para que el aumento de la temperatura mundial se mantenga por debajo de 1,5 °C. Esto limitará los daños. Pero para África y otras regiones vulnerables, este daño ya es grave. Alcanzar el objetivo prometido de proporcionar 100 000 millones de dólares de financiación climática al año es ahora crítico a nivel mundial si queremos prevenir los riesgos sistémicos de dejar a las sociedades en una situación crítica. Esto puede hacerse asegurando que estos recursos se centren en aumentar la resiliencia a los impactos actuales e inevitables de la crisis climática, así como en apoyar a las naciones vulnerables para que reduzcan sus emisiones de gases de efecto invernadero: un balance entre adaptación y mitigación. Estos recursos deben llegar a través de asistencias y no de préstamos, y deben ser incrementados con urgencia antes del actual periodo de revisión de 2025. Estos recursos deben abordar, en primer lugar, la resiliencia de los sistemas de salud, ya que las crisis agravadas por el clima se manifiestan a menudo en problemas sanitarios agudos. Fondos de fomento para políticas de adaptación resultarán más rentables que recurrir a la ayuda en caso de catástrofe.

Ha habido avances en materia de adaptación en África y en el mundo, incluyendo sistemas de alerta temprana y de infraestructura para defenderse de los extremos climáticos. Pero los países de primera línea no reciben compensación por los impactos de una crisis que no han causado. Esto no sólo es injusto, sino que también impulsa la espiral de desestabilización mundial, ya que las naciones invierten dinero en responder a los desastres, pero quedan sin margen para invertir en una mayor resiliencia o en el problema de fondo, que es la reducción de emisiones. Ahora es el momento de introducir mecanismos de financiación para cubrir las pérdidas y los daños, con recursos adicionales que vayan más allá de los destinados a la mitigación y la adaptación. Hay que avanzar y superar los fracasos de la COP26, donde la sugerencia de un mecanismo de este tipo se rebajó a "un diálogo" (18).

La crisis climática es producto de la inacción mundial, y tiene un gran coste no sólo para los países africanos, desproporcionadamente afectados, sino para todo el mundo. África se une a otras regiones de primera línea para instar a las naciones ricas a que den por fin un paso al frente, aunque sólo sea por el hecho de que la crisis en África se extenderá más pronto que tarde y envolverá todos los rincones del planeta, momento en el que puede ser demasiado tarde para responder con eficacia. Si hasta ahora no les han convencido los argumentos morales, esperemos que ahora prevalezca su propio interés.
